# HTLV Tax: A Fascinating Multifunctional Co-Regulator of Viral and Cellular Pathways

**DOI:** 10.3389/fmicb.2012.00406

**Published:** 2012-11-30

**Authors:** Robert Currer, Rachel Van Duyne, Elizabeth Jaworski, Irene Guendel, Gavin Sampey, Ravi Das, Aarthi Narayanan, Fatah Kashanchi

**Affiliations:** ^1^National Center for Biodefense and Infectious Diseases, George Mason UniversityManassas, VA, USA; ^2^Department of Microbiology, Immunology, and Tropical Medicine, The George Washington University Medical CenterWashington, DC, USA

**Keywords:** HTLV-1, Tax, NF-κB, post-translational modification

## Abstract

Human T-cell lymphotropic virus type 1 (HTLV-1) has been identified as the causative agent of adult T-cell leukemia (ATL) and HTLV-1-associated myelopathy/tropical spastic paraparesis (HAM/TSP). The virus infects between 15 and 20 million people worldwide of which approximately 2–5% develop ATL. The past 35 years of research have yielded significant insight into the pathogenesis of HTLV-1, including the molecular characterization of Tax, the viral transactivator, and oncoprotein. In spite of these efforts, the mechanisms of oncogenesis of this pleiotropic protein remain to be fully elucidated. In this review, we illustrate the multiple oncogenic roles of Tax by summarizing a recent body of literature that refines our understanding of cellular transformation. A focused range of topics are discussed in this review including Tax-mediated regulation of the viral promoter and other cellular pathways, particularly the connection of the NF-κB pathway to both post-translational modifications (PTMs) of Tax and subcellular localization. Specifically, recent research on polyubiquitination of Tax as it relates to the activation of the IkappaB kinase (IKK) complex is highlighted. Regulation of the cell cycle and DNA damage responses due to Tax are also discussed, including Tax interaction with minichromosome maintenance proteins and the role of Tax in chromatin remodeling. The recent identification of HTLV-3 has amplified the importance of the characterization of emerging viral pathogens. The challenge of the molecular determination of pathogenicity and malignant disease of this virus lies in the comparison of the viral transactivators of HTLV-1, -2, and -3 in terms of transformation and immortalization. Consequently, differences between the three proteins are currently being studied to determine what factors are required for the differences in tumorogenesis.

## Introduction

The Human T-cell lymphotropic virus type 1 (HTLV-1) was discovered in the early 1980s by two independent groups working in the United States (Gallo lab; Poiesz et al., 1980, 1981) and Japan (Hinuma lab; Yoshida et al., 1982). It is a complex retrovirus and a member of the *Deltaretrovirus* genus. Although there are currently four known types of HTLV, HTLV-1 is by far the most pathogenic of the group and has the distinction of being the first oncogenic retrovirus discovered in humans (Mahieux and Gessain, 2007). It infects an estimated 15–20 million people worldwide and has been implicated as the causative agent in a number of disease conditions. Notably, among these conditions is Adult T-cell Leukemia (ATL) and HTLV-1-Associated Myelopathy/Tropical Spastic Paraparesis (HAM/TSP). HAM/TSP was first described in 1969 over a decade prior to the discovery of HTLV-1. It presents with inflammatory symptoms and incomplete paralysis of the limbs (Gessain et al., 1986; Kfoury et al., 2012). ATL was first characterized by the work of (Takatsuki et al., 1977; Poiesz et al., 1980; Yoshida et al., 1984; Gallo, 2011; Kfoury et al., 2012). It develops in approximately 2–5% of all HTLV-1 infected patients and results in an aggressive disease course that is highly resistant to current chemotherapy treatments. The rates of HAM/TSP are less easily determined as the disease is often misdiagnosed; however, it has been estimated that there are approximately 3,600 unrecognized cases of HAM/TSP in the United States alone (Orland et al., 2003; Goncalves et al., 2010; Poetker et al., 2011).

**Figure 1:**
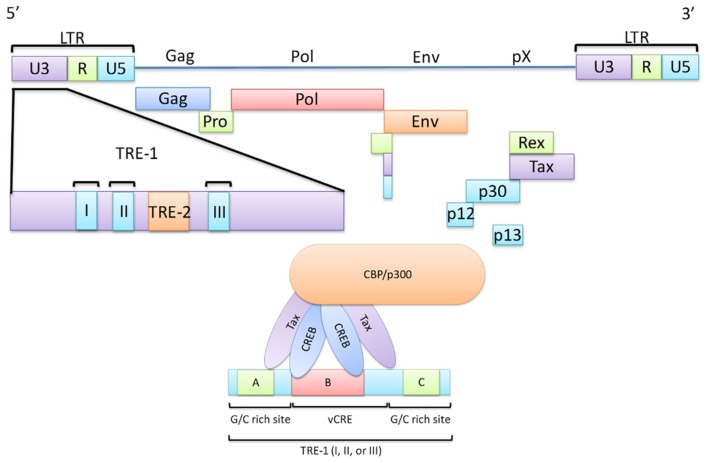
**The HTLV-1 viral genome highlighting the Transcriptional Response Elements (TRE)**. A schematic diagram of the HTLV-1 viral genome illustrating the interaction between the TRE-1 and the Tax/CREB/CBP/p300 complex. Transactivation of transcription at the viral LTR is a critical role of Tax in HTLV-1 infected cells as this ultimately leads to the expression of all viral genes. Here, major viral proteins are linked to their genomic regions and the LTR is divided into the U3, R, and U5 regions. The U3 region is of significant importance in Tax-mediated transcription and thus is highlighted. This region contains three TRE-1 regions, each capable of recruitment of the Tax/CREB/CBP/p300 complex. TRE-1 is shown to contain three domains (A, B, and C) of which B, in conjunction with either A or C, is required for the Tax/CREB/CBP/p300 complex to bind to the LTR.

The HTLV-1 transactivator protein, Tax, has been identified as a protein of significant interest in HTLV-1 pathogenesis as it is a potent activator of a variety of transcription pathways and has been shown to be sufficient to immortalize T-cells *in vitro* and thus plays an important role in cellular transformation (Yao and Wigdahl, 2000; Grassmann et al., 2005; Kashanchi and Brady, 2005; Mahieux and Gessain, 2007). Tax is a highly promiscuous viral protein, coded by open reading frame (ORF) IV in the pX region of the HTLV-1 genome, as seen in Figure 1 (Brady et al., 1987; Yoshida et al., 1989; Yoshida, 1994). The protein is comprised of 353 amino acids (40 kDa) and contains a number of interesting domains that allow it to interact with a myriad of cellular factors thus affecting a large number of cellular functions and products (Harrod et al., 1998; Nicot et al., 1998; Chun et al., 2000; Gachon et al., 2000; Xiao et al., 2000; Li et al., 2003; Hirata et al., 2004; Wu et al., 2004; Kfoury et al., 2012). However, a complete mechanism for Tax-mediated oncogenesis remains to be fully elucidated. In this review, an important set of Tax interactions in HTLV-1 infected cells are summarized. The potential roles of Tax in transcription and oncogenesis are emphasized. Comparisons between the Tax proteins of HTLV-1, -2, and -3 are also highlighted.

## Tax Promotes Transcription by Transactivating Cellular Transcription Factors

The HTLV-1 genome is capped on either end by a long terminal repeat (LTR). Each LTR contains three regions, the unique 3′ (U3), the repeated (R), and the unique 5′ (U5) regions (Figure 1). While the R and U5 regions have been shown to be involved in a number of protein interactions, an interaction with Tax remains to be fully described (Takebe et al., 1988; Seiki et al., 1990; Gartenhaus et al., 1991; Kashanchi et al., 1993; Xu et al., 1996). The U3 region, however, has been shown to be of importance in regard to regulating proviral gene expression as it contains a region known as the Tax responsive element I (TRE-1; Seiki et al., 1983; Yao and Wigdahl, 2000; Jiang et al., 2009). TRE-1 is comprised of three discontinuous 21-basepair (bp) repeats. Only one of these repeats is required for transcription; however, mutations of the middle repeat (TRE-1 II) have been shown to cause the most significant reduction in transcription efficiency (Barnhart et al., 1997). Each repeat contains three conserved domains (termed A, B, and C) which comprise a total of 13 nucleotides out of the total 21 (Montminy et al., 1986; Montminy and Bilezikjian, 1987; Montagne et al., 1990; Yao and Wigdahl, 2000). Domain B is the most important in terms of Tax-mediated transactivation as it contains five of the eight bp that make up what is known as the viral cAMP response element (vCRE) and, in conjunction with either domain A or C, domain B is sufficient for Tax-mediated transactivation. vCRE is the site at which Tax initiates transcription from the viral promoter. This is achieved by successful binding of a protein complex made up (at least in part) by the cAMP response element binding protein (CREB), Tax, and the CREB-binding protein (CBP), or p300. Below each of these proteins are discussed in their relation to Tax-mediated transcription.

### CREB is specific for vCRE activation in the presence of Tax

cAMP response element binding protein interacts directly with the vCRE, thus making it a protein of paramount importance in Tax-mediated transactivation of the viral promoter (Montminy et al., 1986; Montminy and Bilezikjian, 1987; Montagne et al., 1990; Yao and Wigdahl, 2000). CREB is recruited by Tax where it dimerizes on the vCRE region of TRE-1 forming either a CREB homodimer or CREB-Activating transcription factor family (ATF) protein heterodimer through interaction between the leucine zipper domains of CREB/ATF. Tax forms a homodimer by linkage between its zinc finger domains and binds to the CREB dimer (Franklin et al., 1993; Habener et al., 1995; Yao and Wigdahl, 2000). The Tax dimer associates with the G and C rich sequences that flank the vCRE thus lending enhanced DNA binding specificity to the CREB dimer. The resulting protein complex is specific to TRE-1 and is vital for efficient continuous transcription at the vCRE (Wagner and Green, 1993; Anderson and Dynan, 1994; Paca-Uccaralertkun et al., 1994; Tie et al., 1996). The ultimate binding, however, of the Tax/CREB complex to the vCRE is accomplished through the use of three tandem domains located on the CREB protein: a leucine zipper domain in the carboxyl terminus, a transactivation domain in the amino terminus, and a basic DNA binding domain adjacent to the leucine zipper domain (Hoeffler et al., 1988; Gonzalez et al., 1989; Habener et al., 1995; Yao and Wigdahl, 2000). This formation of the Tax/CREB/vCRE complex comprises a vital first step in transcriptional activity at the LTR. It is important to note however, that while the Tax/CREB/vCRE complex forms in a well characterized fashion, a complete description of all associated factors still remains to be developed.

Transducers of regulated CREB (TORC) proteins are an interesting example of one of these associated host factors. TORCs enhance the interaction of CREB with the TFII130 component of Transcription factor II D (TFIID) thus enhancing the transcriptional initiation potential of CREB (Koga et al., 2004; Siu et al., 2006). These proteins can also interact with Tax to enhance HTLV-1 transcription (Koga et al., 2004; Siu et al., 2006; Nyborg et al., 2010). The work of Jiang and coworkers has proposed an interesting potential role for TORC2 in HTLV-1 infections. Using a Tax – GFP construct which is suppressed *in vivo* but upregulated *ex vivo*, thus emulating latent infections, they showed that the downregulation of TORC2 corresponded with modeled latent infections as compared to modeled acute infections (Jiang et al., 2009). This observation is intriguing as it suggests that the Tax/CREB interaction may play an integral role in the distinction between latent and acute infections and thus further underscoring the critical role of CREB in HTLV-1 infections.

### CBP/p300 interacts with Tax to promote transcription by chromatin remodeling

Another critical factor in HTLV-1 infections is the recruitment of CBP/p300 to the Tax/CREB complex. CBP and p300 constitute a pair of large (approximately 300 kDa) coactivator proteins that share a high degree of homology (Kwok et al., 1996; Laurance et al., 1997; Harrod et al., 2000; Chan and La Thangue, 2001; Lu et al., 2002). They are involved in the regulation of almost all known pathways of gene expression in multicellular organisms. The ability of CBP/p300 to stimulate transcription is largely due to their ability to acetylate both histone and non-histone substrates (Zhang et al., 2008). Specifically, histone hyperacetylation has been shown to be strongly associated with active transcription. Upon recruitment to the viral promoter, the CBP/p300 complex promotes chromatin remodeling (Kashanchi et al., 1998; Sharma and Nyborg, 2008; Nyborg et al., 2010). This observation lends strong support to the idea that Tax promotes such high levels of transcription by promoting conformational changes in the chromatin structure of the host cell.

Much like the interaction of CREB with Tax, the chromatin remodeling required for Tax-mediated transactivation is not the function of a single protein but rather the concert interactions of many polypeptides. Of particular interest in chromatin remodeling complexes is the SWI/SNF (switching-defective-sucrose non-fermenting) complex. Through a combination of chromatography and immunoprecipitation techniques, the work of Easley et al. (2010) has demonstrated that Tax interaction with CBP/p300 and SWI/SNF complexes are closely associated in HTLV-1 infected cells. Utilizing a series of Brahma-related gene 1 (BRG1) mutants and siRNA induced knockdown cells, it has been reported that the SWI/SNF complex PBAF is vital for Tax activated transcription of the integrated HTLV-1 genome. This assertion is supported by the work of Van Duyne et al. (2011), who also demonstrated a strong expression of Baf170 (a component of the PBAF complex) in HTLV-1 infected cell lines where very little Baf170 was detected in uninfected cell lines. Easley et al. further confirmed a decrease in histone density around the LTR after one round of Tax activated transcription. This observation correlates well with the chromatin remodeling capabilities of the SWI/SNF complex and lends further support to the hypothesis that Tax regulates transcription at both the level of recruitment of chromatin remodeling complexes and the level of recruitment of transcriptional factors. Evidence was provided to suggest that the addition of Tax to the SWI/SNF complex could increase the rate of chromatin remodeling of the nucleosome thus aiding in the function of CBP/p300 (Easley et al., 2010). Collectively, these data reveal CBP/p300 as a vital factor in both Tax-mediated transactivation and Tax-mediated chromatin remodeling.

### Chromatin remodeling is of vital importance to Tax-mediated transactivation

Further work examining the proteins associated with Tax-mediated transcription of the HTLV-1 LTR suggests that chaperone molecules such as Nucleosome assembly protein 1 (NAP1) may be involved in Tax-mediated chromatin remodeling. NAP1 is a histone chaperone molecule that has been shown to be involved in transcription-independent nucleosome eviction at the HTLV-1 promoter (Sharma and Nyborg, 2008). These colleagues demonstrated this by assembling chromatin templates in the absence of assembly proteins via salt deposition. The resultant chromatin was shown to be indistinguishable from chromatin formed via assembly factors; however, a subsequent DNA pulldown assay revealed that the presence of p300 and Acetyl-CoA were no longer sufficient for nucleosome eviction. The authors then introduced purified NAP1, effectively rescuing nucleosome eviction from the chromatin template (Sharma and Nyborg, 2008). This nucleosome eviction activity, however, was shown to be dependent on the HAT-activity of CBP/p300. The eviction process removes intact histones from the chromatin structure thus opening the promoter for transcription. These data support the emerging hypothesis that acetylation-dependent promoter nucleosome disassembly is a pre-requisite for strong transcriptional activation.

In conjunction with this previous observation, p300 recruitment to an integrated HTLV-1 promoter has been shown to correlate with a decrease in acetylated histones (Lemasson et al., 2006; Bogenberger and Laybourn, 2008; Nyborg et al., 2010). This is unexpected as the function of p300 is to acetylate histones at the site of the promoter. However, this observation coincided with a similar reduction in histone H3 and linker histone H1 levels. Together, these observations would suggest that there was a reduction in nucleosome density at the promoter (Lemasson et al., 2006; Bogenberger and Laybourn, 2008; Nyborg et al., 2010). This supports the supposition that Tax induces nucleosome eviction at the promoter, thus enabling strong transcriptional activity at the HTLV-1 promoter. In fact, global reduction in histone levels in HTLV-1 infected cell lines has been reported (Bogenberger and Laybourn, 2008; Sharma and Nyborg, 2008). Furthermore, Bogenberger and Laybourn (2008) reported that Tax alone is sufficient to reduce histone transcript levels in Jurkat cells. Combined with the reported ATP-independent chromatin remodeling capabilities of NAP1, this observation of histone reduction in Tax expressing cells suggests that the presence of Tax results in a prolonged unfolding of the chromatin structure; therefore, allowing constant transcriptional activation. Thus, these data begin to describe a possible mechanism for the strong transcriptional activity of factors activated by Tax.

In contrast to the HAT/chromatin unfolding activity of Tax associated factors, work with histone deacetylase complexes (HDACs) has demonstrated their ability to counteract the action of HAT-activity containing factors. When overexpressed, HDACs interact directly with Tax and bind to the HTLV-1 promoter, thus repressing transcription by refolding chromatin fibers (Ego et al., 2002; Lemasson et al., 2002, 2004; Lu et al., 2004; Mosley et al., 2006). Consequently, they serve as a counterbalance to otherwise largely unchecked Tax-mediated transcriptional activation. Thus it is not surprising that Tax excludes HDAC activity at the LTR (Mosley et al., 2006). This exclusion combined with the recruitment of HAT-activity containing factors, such as CBP/p300, lends even further support to the idea of Tax promoting a constant “open” state of the chromatin structure.

Notably, the work of Hieshima et al. (2011) has recently added musculoaponeurotic fibrosarcoma oncogene homolog, c-Maf (a protein that can function as a transcriptional activator or repressor depending on its binding) to the list of potentially significant transcription factors in terms of inhibition of the Tax/CBP/p300 interaction. Using a series of luciferase assay based experiments, they have reported that c-Maf inhibits Tax-dependent promoter activation by competing with Tax for binding to the zinc finger domain of CBP (Hieshima et al., 2011). The observations regarding HDACs and c-Maf described above not only underscore the importance of CBP/p300 in Tax-mediated transcription of the LTR, but also provide two potential avenues of downregulating viral transcription.

## Tax Promotes the Transcription of Cellular Proteins by Activating Several Cellular Factors

Though the transactivation of the LTR is an essential role of Tax, it is not the only role of this fascinating oncoprotein. It also acts upon the host cell to regulate, manipulate, and exploit host cellular pathways to mediate cellular transformation. Below, several key transcriptional factors are discussed in their relation to their interaction with Tax.

### The serum response factor is activated by Tax

The serum response factor (SRF) is one of the major cellular proteins activated by Tax. Genes such as *c-fos, Erg-1, Erg-2, Fra-1, c-Jun*, and *JunD* all have SRF binding sites in their respective promoters and Tax has been shown to activate all of these genes (Suzuki et al., 1993; Fujii et al., 1994, 1995; Winter and Marriott, 2007). This suggests Tax has an involvement in regulating SRF-dependent transcription. Furthermore, Tax activation of this factor has been linked to binding of the serum response element (SRE). As a number of growth regulatory genes are responsive to SRF signaling, Tax may utilize this pathway to manipulate cell cycle and consequently contribute to cellular transformation. Interestingly, c-*fos* has been shown to be a potent cellular oncogene, further suggesting a link between Tax manipulation of SRF-mediated transcription and oncogenesis (Suzuki et al., 1993; Fujii et al., 1994, 1995; Winter and Marriott, 2007).

The DNA sequence CC(A/T)_6_GG (termed the CArG box) when situated next to a transcription factor family (TCF) Ets element comprise the SRE. The SRF and TCF (members of which are part of the Ets family of proteins) proteins bind to the SRE creating a ternary complex at the promoter. Tax has been shown to interact with members of the TCF family, Elk-1 and SAP-1, and thus by interacting with the TCF protein of this ternary structure, Tax may dysregulate the SRF pathway (Suzuki et al., 1993; Fujii et al., 1994, 1995; Winter and Marriott, 2007). Moreover, HTLV-1 has been shown to contain a CArG box of its own (vCArG) within its own SRE (vSRE). This vSRE is located within the TRE-2 region of the U3 region of the LTR. The work of Winter and Marriott has demonstrated that not only does Tax interact with SRF directly but that in the presence of Tax, the SRF protein recognizes and binds to a more diverse group of sequences as compared to the absence of Tax. Also, increased binding of SRF to the c-*fos* promoter in Tax expressing cells versus non-Tax expressing cells is documented (Winter and Marriott, 2007). The continued characterization of the interaction between Tax and SRF has provided further evidence of dysregulation of cellular growth by Tax-mediated methods (further discussed in later sections) and continues to provide mechanisms by which Tax immortalizes cells and initiates the oncogenic progression of cells.

## Tax-Mediated Activation of the NF-κB Pathway is Vital for HTLV-1 Transformation

It is well established that Tax interacts with the host transcription factor NF-κB, resulting in the activation of the NF-κB pathway, which is critical for transformation, proliferation, and survival of HTLV-1 infected cells. Due to the significance of Tax/NF-κB interaction on HTLV-1 disease states, NF-κB is given special focus in this review.

### NF-κB is a family of transcription factors

The NF-κB family of transcription factors variably regulates gene expression as a response to dynamic post-translational modifications (PTMs), subcellular localization, and formation of homo and heterodimer complexes of the family members (Ghosh et al., 1998; Silverman and Maniatis, 2001; Karin and Lin, 2002; Ruland and Mak, 2003; Sun and Xiao, 2003). This family of transcription factors consists of five structurally similar DNA-binding proteins, RelA (p65), RelB, c-Rel, p50/NF-κB1, and p52/NF-κB2 (Siebenlist et al., 1994). Closely related is the IκB family which contains p100 and p105 (the two precursor proteins for the NF-κB family members p50 and p52), respectively; the typical IκB proteins, IκBα and IκBε; and the atypical IκB proteins, BCL-3, IκBβ, IκBδ, and IκBNS (Siebenlist et al., 1994). The NF-κB proteins tend to segregate in the cytoplasm of unstimulated cells, forming inactive heterodimers with IκB proteins. Due to the innate differences in activation mechanisms, two distinct NF-κB pathways exist, the canonical pathway, which is involved in the regulation of inflammation and apoptosis, and the non-canonical pathway, which regulates lymphoid organogenesis, to name a few cellular processes (Lo et al., 2006; Shembade and Harhaj, 2010). The NF-κB canonical pathway is activated by intra- and extracellular stimuli, including the penultimate NF-κB activator, the pro-inflammatory cytokine TNF-α, as well as cellular oxidative stress or the presence of virions and bacterial virulence factors. These extracellular stimuli bind to membrane-bound receptors spanning from the cytoplasm to the extracellular space and include Growth Factor Receptors (GFRs), TNF-α Receptor (TNFR), IL-1 Receptor (IL1R), Toll Like Receptors (TLRs), T-cell Receptors (TCRs), and B-cell Receptors (BCRs). The non-canonical NF-κB pathway is activated by a smaller subset of proteins as compared to the canonical pathway, specifically, the TNF superfamily members such as B-cell activating factor, CD40 ligand, CD70, and the receptor activator of NF-κB (RANK) ligand. Both pathways, however, ultimately involve the activation of the IKK (IκB kinase) complex composed of two catalytic subunits, IKKα (IKK1) and IKKβ (IKK2), and one regulatory subunit, IKKγ (NEMO). The activation of IKK, results in the phosphorylation of the inhibitory IκBs, rendering them inactive. This inactivation event allows the completion of the pathway, resulting in nuclear translocation and gene expression.

### Tax mediates the activation of both the canonical and non-canonical NF-κB pathways

The activation of the NF-κB pathway is highly regulated in normally dividing cells; however, most cancerous cells exhibit dysregulation of this pathway, often in a constitutively active state. This activation is observed in HTLV-1 infected, transformed T-cells as well as in ATL cells due primarily to the stimulation of both the canonical and non-canonical pathway by Tax (Hirai et al., 1992; Suzuki et al., 1993, 1994, 1995; Beraud et al., 1994; Lanoix et al., 1994; Murakami et al., 1995; Petropoulos and Hiscott, 1998; Yoshida, 2001).

Tax activates the canonical pathway through multiple interactions with cellular proteins. In the cytoplasm, Tax directly interacts with the IKK regulatory subunit IKKγ, resulting in the phosphorylation, ubiquitination, and degradation of IκB and subsequent phosphorylation, activation, and nuclear translocation of RelA (Chu et al., 1998, 1999; Harhaj and Sun, 1999; Jin et al., 1999; Sun et al., 2000; Xiao and Sun, 2000; Xiao et al., 2000; Carter et al., 2001b). The interaction of Tax with IKKγ has been demonstrated *in vitro* in both HTLV-1 transfected cells, as well as HTLV-1 transformed cell lines. Supporting this observation, a lack of Tax-mediated NF-κB activation is seen in cells deficient in IKKγ. In the nucleus, Tax recruits and manipulates RelA into subcellular foci, known as Tax nuclear bodies (Tax NBs), where NF-κB transcriptional activation is at a maximum level of activity (Semmes and Jeang, 1996; Bex et al., 1997). The interaction between Tax and the IKK catalytic subunits (α and β) remains to be elucidated; however it is suggested that Tax can self-dimerize, bringing different IKK components in close proximity, therefore promoting IKK complex cross-phosphorylation (Tie et al., 1996; Jin and Jeang, 1997; Xiao and Sun, 2000; Qu and Xiao, 2011). Another possibility is that Tax recruits upstream factors, such as TGF-β (transforming growth factor beta), MAP3K (mitogen activating protein kinase kinase kinase), MEKK1 (MEK kinase 1), NIK (NF-κB-inducing kinase), Tpl2, and TAK1 (TGF-β activated kinase 1) to activate the IKK complex (Uhlik et al., 1998; Yin et al., 1998; Yu et al., 2008). Recently, a cell-free assay system was developed by Shibata et al. (2011) to analyze Tax-induced IKK activation. This system showed that IKK could not be directly activated by Tax, however was activated by MEKK1, and also suggested that Tax-K63-linked polyubiquitination is necessary for Tax-induced IKK activation (Shibata et al., 2011). Another study suggested that Tax can bind directly to IKKγ and activate IKK independent of the signaling pathways physiologically induced by cytokines (Shimizu et al., 2011). Tax activates the non-canonical pathway by directly inducing the processing of p100 to p52 through the recruitment of a IKKγ/IKK complex containing only IKKα, which both activates and recruits IKKα onto the p100 complex. Once phosphorylated, p100 is ubiquitinylated and processed by the proteasome as in the physiological process. This Tax-mediated activation occurs in the absence of NIK. Tax has also been shown to relocalize IKK to subcellular locations, such as the centrosome, ER, Golgi-associated structures, the perinuclear compartment, or lipid raft microdomains as a new locale for Tax-mediated IKK activation.

### NF-κB is strongly activated in HTLV-1 infected cells

**Figure 2:**
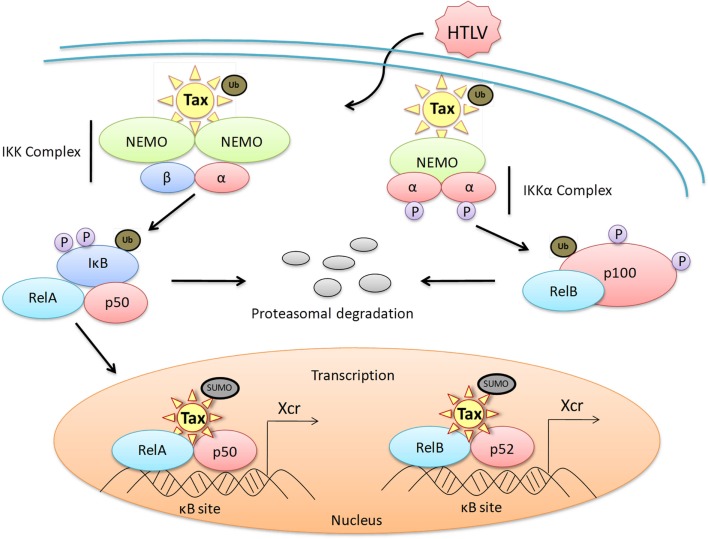
**Dysregulation of the NF-κB canonical and non-canonical pathways by HTLV-1 Tax**. A representative diagram of the protein-protein interactions of HTLV-1 Tax with members of the NF-κB family of transcription factors both in the cytoplasm and the nucleus of an HTLV-1 infected cell. Dysregulation of the canonical pathway occurs with the interaction of ubiquitinated Tax to the cytoplasmic IKK complex, specifically binding to the IKKγ subunit. This interaction results in the phosphorylation of IκB, as well as the ubiquitination and subsequent degradation of IκB through the proteasome pathway. RelA is subsequently activated and translocates into the nucleus where sumoylated Tax recruits RelA to Tax nuclear bodies, driving Tax-mediated NF-κB transcription. Dysregulation of the non-canonical pathway occurs when biquitinated Tax interacts with the IKKα complex and activates the processing of the precursor proteinp100 to p52. This promotes the recruitment of RelB to the nucleus for activation of Tax-mediated NF-κB transcription. Xcr, transcription.

The NF-κB pathways are regulated with multiple feedback loops and modulated by endogenous proteins, resulting in rapid, efficient, and transient cellular gene expression. Like most cancers and viral infections, HTLV-1 infection coincides with a loss of the NF-κB pathway control mechanisms, resulting in constitutive activation of the cascade in both HTLV-1 cell lines and primary ATL cells. Interestingly, this activation does not occur through overstimulation of the T-cell receptor (TCR) or other cellular signaling proteins and kinases. As seen in Figure 2, Tax directly accomplishes the activation of the pathway by binding to p105, p50, p65, and p100 preventing binding of IκB inhibitors and stabilizing the protein, as well as directly binding to IKK, resulting in constant degradation of IκBα (Hirai et al., 1992; Suzuki et al., 1993, 1994; Lacoste et al., 1994; Murakami et al., 1995). Tax promotes the robustness of this pathway by inducing the overexpression of NF-κB proteins, stimulatory cytokines, and receptors as well as preventing the termination of the pathway. Specifically, Tax has been shown to inactivate the NF-κB negative regulatory protein A20 (TNFAIP3) by blocking the assembly of the A20 ubiquitin-editing complex through the inhibition of TAX1BP1 phosphorylation. This prevents the ubiquitination of critical signaling proteins, such as TRAFs, for degradation (Lee et al., 2000; Wertz et al., 2004; Shembade et al., 2007a, 2008, 2009; Shembade and Harhaj, 2010). A20 is a member of a NF-κB negative regulation, ubiquitin-editing complex containing other regulatory proteins such as TAXIBP1, Itch, and RNF11 (Shembade et al., 2008). Additionally, Tax is dependent on the E2 ubiquitin-conjugating enzyme Ubc13 to interact with IKKγ, and resultant Tax-mediated NF-κB activation. The overall activation of NF-κB in HTLV-1 infected cells is persistent regardless of Tax expression in transformed cells.

## Tax Both Undergoes and Promotes Post-Translational Modification

As with most cellular proteins, virally encoded proteins utilize PTMs to modify their enzymatic efficiency and expand their range of functionality. Modifiers allow viruses to carry out a diverse set of effects in the host cell with only a limited number of expressed proteins. In HTLV-1 infected cells, the virally encoded Tax protein is modified by numerous PTMs. Additionally, Tax acts upon host cell proteins to alter their PTM moieties. The modifiers taken on and generated by Tax allow it to affect numerous reactions within the host cell that are vital to the viral life cycle and also lead to many of the pathogenic outcomes indicative of HTLV-1 infections. Here we examine in detail the Tax-induced PTMs of host cell proteins, as well as review known PTMs of Tax and the mechanisms by which they are generated.

### Tax modifies the IKK complex

Several key PTMs of host cell proteins induced via interactions with the HTLV-1 Tax protein have been elucidated. One vital regulatory complex that takes on numerous PTMs due to its association with Tax is the IKK complex. It has been shown that Tax can associate with all three subunits of the IKK, but that its interaction with the IKKγ subunit leads to IKKβ and IKKγ phosphorylation (Chu et al., 1998; Carter et al., 2001a). The related IKKβ phosphorylation has further been characterized to the Ser177 and Ser181 residues within the T-loop of the protein (Carter et al., 2001a). Moreover, after phosphorylation of IKKβ at the Ser177 and Ser181 sites through the association of the IKK complex with Tax, IKKβ is then mono-ubiquitinated. This subsequent ubiquitination of IKKβ is required for biological activation of the IKK complex (Carter et al., 2003). Furthermore, the mechanism by which IKK phosphorylation is established has also been elucidated. Specifically, it has been demonstrated that Tax actually simultaneously associates with IKK and the TGF-β activated kinase 1 (TAK1) and stimulates TAK1 to phosphorylate IKK thereby leading to activation of the complex (Wu and Sun, 2007). Overall, the induced IKK PTMs lead to persistent activation of IKK and phosphorylation of the IKK target, IκBα, at Ser32 and Ser36, which in turn leads to ubiquitin-proteasome degradation (Chu et al., 1998).

### Tax modifies a number of host cell proteins

In addition to the PTMs induced by Tax on the IKK complex, there are several other host cell proteins that are modified due to their association with Tax. For one, Tax mediates the interaction between phosphorylated FoxO4, a tumor suppressor, and the E3 ubiquitin ligase MDM2. This Tax-mediated recruitment of FoxO4 to MDM2 leads to polyubiquitination of FoxO4 and its proteasomal degradation (Oteiza and Mechti, 2011). Additionally, Tax can recruit chromatin remodelers and thereby stimulate epigenetic modifications at the integrated viral genome, which leads to varied viral transcription. The coactivator-associated arginine methyltransferase 1 (CARM1) and Tax associate together leading to increased HTLV-1 transactivation via methylation of histone H3 (Jeong et al., 2006). Alternately, histone methyltransferase SUV39H1 can be tethered to the HTLV-1 LTR through an interaction with Tax that leads to repressed transactivation through methylation of H3K9 (Kamoi et al., 2006). As evident with these opposing enzymatic activities (i.e., CARM1 and SUV39H1), there needs to be vigorous, future research to further define the function of these Tax interacting proteins which may control LTR or cellular gene expression.

### Tax undergoes ubiquitination and SUMOylation

Two well characterized PTMs of Tax are ubiquitination and SUMOylation. These PTMs are used by the cellular machinery to translocate Tax to various subcellular compartments and are also required for the interaction of Tax with various host cell proteins. Notably, PTMs of Tax are required for Tax-mediated activation of the NF-κB pathway. It has been shown that Tax is ubiquitinated through its interaction with Ubc13 (Shembade et al., 2007b). DNA damage stimulates the monoubiquitination of nuclear Tax at Ser280 and Ser284 which drives its subsequent export from the nucleus (Gatza et al., 2007). Once ubiquitinated, Tax associates with the IKK complex in the cytoplasm and drives nuclear translocation of the RelA component of the NF-κB cascade. SUMOylated Tax associates with RelA, p300, and free IKKγ in nuclear bodies. It has been shown that both Lys280 and Lys284 are required for proper SUMOylation of Tax, while lysine residues 189, 197, 263, 280, and 284 are all targets for ubiquitination (Lamsoul et al., 2005; Nasr et al., 2006). It has also been shown that the SUMO-dependent ubiquitin ligase, Really Interesting New Gene Finger Protein 4 (RNF4), binds to SUMOylated Tax next to the Lys280/Lys284 ubiquitin and SUMO modification sites and ubiquitinates the nuclear located protein. The ubiquitination of Tax by RNF4 leads to the cellular translocation of Tax to the cytoplasm and subsequently increases NF-κB activation while decreasing Tax activity associated with interaction with nuclear CREB (Fryrear et al., 2012). Ubiquitination of Tax at lysine residues 263, 280, and 284 is also required for Tax assisted localization of IKKγ to the Golgi (Harhaj et al., 2007). Interestingly, ubiquitination of these three sites results in Tax interaction with the proteasome; however, it is not degraded via the ubiquitin-labeled recruitment to the proteasome (Chiari et al., 2004). Furthermore, the association of Tax and IKKγ within the Golgi has been shown to be mediated by an additional interaction with NEM-related protein (NRP/optineurin). The generation of the Tax and NRP complex was shown to be driven by the binding of polyubiquitinated Tax to the ubiquitin-binding domain of NRP. Moreover, this interaction was also shown to stabilize the ubiquitinated Tax and to increase activation of the NF-κB pathway. Additionally, the Tax binding protein, TAX1BP1, was also demonstrated to be necessary for the Tax and NRP complex, therefore indicating a functional NF-κB activating multimeric complex consisting of these three proteins (Journo et al., 2009). Another recent report showed that bonding through the Lys63 residue of the ubiquitin protein is necessary for Tax-dependent activation of IKK while linear polyubiquitination of Tax is not required for IKK activation (Shibata et al., 2011).

Acting in opposition to Tax ubiquitination, the deubiquitinase CYLD interacts with Tax and removes ubiquitin moieties thereby reducing the association of Tax with IKK. However, within HTLV-transformed cells, CYLD is constitutively phosphorylated which inhibits its deubiquitinase activity and, therefore, Tax association with IKK is maintained (Wu et al., 2011). Another deubiquitinase, Ubiquitin-specific peptidase 20 (USP20), was also shown to reduce Tax ubiquitination and consequently inhibit Tax-mediated upregulation of the NF-κB pathway. Similar to CYLD, though, USP20 is found to be down regulated in HTLV-1 transformed cells although the mechanism of the USP20 suppression has not been elucidated (Yasunaga et al., 2011).

### Tax can also undergo acetylation and phosphorylation

Beyond ubiquitination and SUMOylation, there are other important PTMs that alter the functionality of Tax. One such identified PTM is the acetylation of Tax at Lys346 by p300 in the nucleus which boosts NF-κB dependent transcription (Lodewick et al., 2009). Additionally, phosphorylation of Tax at varying residues has also been shown to modify Tax function and localization. Specifically, when Tax is phosphorylated at either residues Ser300 or Ser301 it is localized in nuclear bodies and aids in the activation of ATF/CREB and NF-κB regulated genes (Bex et al., 1999). Moreover, the pleiotropic serine/threonine kinase CK2 has been shown to phosphorylate Tax at three residues near its PDZ binding domain, specifically at Ser336, Ser344, and Thr351. Of these phosphorylation sites, Thr351 was shown to be requisite for Tax-1 binding to the tumor suppressor scaffold protein hDlg but not for transactivation (Bidoia et al., 2010).

## The Localization of Tax in the Host Cell Determines Function

**Figure 3:**
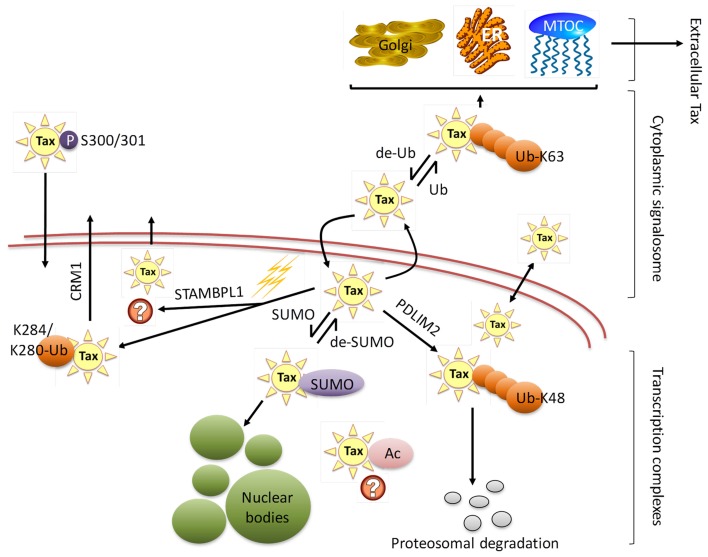
**HTLV-1 Tax subcellular localization**. HTLV-1 Tax undergoes extensive post-translational modifications that regulate Tax trafficking through the cytoplasm and nucleus, binding partners, and consequently its function. The figure shows Tax translocation from nucleus to cytoplasm through a sumoylation/ubiquitination cycle. Cytoplasmic ubiquitinated Tax activates several signal transduction pathways and can be found in association with the endoplasmic reticulum (ER), Golgi apparatus, and microtubule organizing center (MTOC). Additionally, cytoplasmic Tax can be secreted by extracellular shuttling. De-ubiquitinated Tax migrates to the nucleus where it is sumoylated and acetylated, resulting in assembly to nuclear bodies. Nuclear Tax can be involved in a number of processes, including interactions with nuclear proteins, incorporation into nuclear bodies as foci for high transcriptional activity, being targeted for proteasomal degradation, as well as actively being translocated into the cytoplasm via CRM1 pathway or other pathways that need yet to be defined (STAMBPL1). Lysine 48 and 63 ubiquitin branching is denoted as Ub-K48 and Ub-K63 respectively, while monoubiquitination at lysines 280 or 284 is indicated as K284/K280-Ub.

Intracellular trafficking of Tax, which is known to function in both the nucleus and cytoplasm of HTLV-1 immortalized T-cells, is essential for Tax-induced pleiotropic effects and HTLV-1’s ability to successfully modulate transcription and effect transformation (Figure 3). Major progress in the elucidation of Tax nucleo-cytoplasmic shuttling and its associated functionalities has been performed, though the precise regulatory mechanisms of intracellular Tax distribution requires further clarification (Alefantis et al., 2005a; Bertazzoni et al., 2011).

### Nuclear localization of Tax and transcriptional activation

Harboring a nuclear localization signal (NLS) at amino acids 18–52 of its N-terminus, Tax is predominantly a nuclear protein (Smith and Greene, 1992). Nuclear Tax was initially identified as Tax speckled structures (TSS) in HTLV-1 transformed T lymphocytes or cells transfected with Tax expression vectors (Semmes and Jeang, 1996). These discrete foci were later defined as Tax NBs (Bex et al., 1997). Embedded in the central domain of Tax is a nuclear export sequence (NES) at amino acids 188–202, overlapping a region rich in leucine residues (Alefantis et al., 2003). When fused to GFP, recombinant NES was found to direct nuclear export via the CRM1 pathway but this effect was not observed in HTLV-1 infected cells. This is suggestive of full-length Tax utilizing an alternative nuclear export pathway and that the availability of the NES and NLS may require PTMs of Tax, leading to conformational change or macromolecular interactions. Separately, Tax homodimer formation has been shown to be critical for nuclear transport, where Tax proteins deficient in dimerization failed to localize to the nucleus and localization was subsequently rescued once Tax dimerization activity was restored (Jin and Jeang, 1997; Fryrear et al., 2009). Tax dimerization was found to be dictated by a central region encompassing amino acids 127–228, which can be further divided into three sub-dimerization domains (DD) as DD1 (amino acids 127–146), DD2 (amino acids 181 to 194), and DD3 (amino acids 213–228) subdomains (Basbous et al., 2003b). These findings are indicative of a complex interplay between primary and secondary structure, and cellular localization. Moreover, an additional Tax domain was identified to play a role in specific Tax sub-nuclear targeting (Fryrear et al., 2009). The TSS localization signal (TSLS) region spanning amino acids 50–75 was shown to be necessary and sufficient in directing Tax to the TSSs. Interestingly, the TSLS is a distinct and independent regulatory domain from the adjacent Tax NLS.

Post-translational modifications of the Tax protein provide an additional layer of complexity in the regulation of the protein’s cellular distribution, and the correct pattern of these specific PTMs is known to influence Tax nuclear localization. Correspondingly, it has been suggested that unmodified Tax has the ability to freely undergo nucleo-cytoplasmic shuttling (Lamsoul et al., 2005). For example, the interplay between ubiquitination and sumoylation play a major role in the NF-κB stimulating activity of Tax and partake in Tax subcellular localization (Kfoury et al., 2012). This balance between ubiquitination and sumoylation on overlapping C-terminal lysine residues determine the separation of Tax into the nuclear and cytoplasmic compartments, where SUMOylated Tax predominantly exists in the nucleus while ubiquitinated Tax predominantly localizes in the cytoplasm (Gatza et al., 2007). Amongst other functions induced by ubiquitination of Tax, interaction with the ubiquitin E3 ligase PDLIM2 has been shown to direct K48-linked polyubiquitination of Tax which in turn, targets the molecule to the nuclear matrix for protesomal degradation (Yan et al., 2009). Correspondingly and in response to various genotoxic or cellular stress, Tax monoubiquitination of lysines at positions 280 and 284 results in its CRM1-dependent nuclear export (Gatza et al., 2007). Similarly, attachment of SUMO-1 to Tax regulates its localization in NBs, and polysumoylated Tax is localized and detected only to these structures. Sumoylation is believed to regulate retention of Tax in the nucleus (Lamsoul et al., 2005). Likewise, data from Françoise Bex’s group has shown that the acetylated form of Tax can be detected in Tax expressing cells as well as in HTLV-1 transformed T lymphocytes, an event that has transcriptional activation repercussions. This form of modified Tax and the acetylation effecter, p300, are detected to be localized to the nucleus (Lodewick et al., 2009, 2011). Alternatively, phosphorylation of Ser 300 and 301 are critical for the Tax phosphoprotein nuclear migration (Lodewick et al., 2011). A recent RNAi study has identified the metalloprotease STAM-binding protein-like 1 (STAMBPL1) as a host protein regulating Tax nucleo-cytoplasmic transport. STAMBPL1 is a de-ubiquitinating enzyme that stabilizes Tax by preventing its proteasomal degradation in the nucleus (Lavorgna and Harhaj, 2012). In addition, the enzyme is a required factor for DNA damage-induced Tax nuclear export, exemplifying how Tax localization is a dynamic event following DNA damage (Gatza et al., 2007; Lavorgna and Harhaj, 2012). An additional step for Tax re-localization into the nucleus involves interactions with nucleoporin proteins within the nuclear pore complex (NPC) structures (Tsuji et al., 2007). Specifically, interaction of the Tax N-terminal zinc finger domain with p62 nucleoporin was observed and this import event occurs in an energy- and carrier protein-independent manner.

### Cytoplasmic localization of Tax and manipulation of host cellular pathways

As previously discussed, presence of Tax in the cytoplasm and in the nucleus may be required for the activation of Tax-mediated gene expression (Bex and Gaynor, 1998). Overexpression of Tax results in a heterogeneous intracellular Tax distribution that can be diffused, punctuated, or localized in distinct Golgi apparatus-associated lipid raft microdomain structures in the cytoplasm (Lamsoul et al., 2005; Huang et al., 2009; Avesani et al., 2010; Lodewick et al., 2011). The microtubule organizing center (MTOC), in association with the cis-Golgi, has been described as an exclusive extranuclear Tax localization site, in conjunction with the cell–cell contact region that organizes the viral synapse between an infected and uninfected target cell (Nejmeddine et al., 2005). Additionally, Tax has been detected in association with TAB2-containing cytoplasmic foci (Lamsoul et al., 2005; Alefantis et al., 2007; Avesani et al., 2010) and with the centrosome structure (Kfoury et al., 2008).

As indicated previously, the PTM status of Tax has a significant outcome on its functionality and localization. To further illustrate this point, SUMOylation and ubiquitination compete for the same overlapping lysine residues that result in opposing regulatory effects. For example, colocalized Tax and ubiquitin molecules have been detected exclusively in the cytoplasm with subsequent poorly ubiquitinated Tax detection in the nucleus possibly due to rapid proteosomal degradation or cytoplasmic shuttling (Lamsoul et al., 2005; Gatza et al., 2007). It is believed that ubiquitination controls Tax retention in the cytoplasm (Lamsoul et al., 2005). Other PTM dynamics associated with cytoplasmic Tax include Tax phosphorylation as a pre-requisite for cytoplasmic ubiquitination of the protein, in addition to acetylation and sumoylation (Lodewick et al., 2009, 2011). Similar to Tax nuclear import conditions, Tax export into the cytoplasm has been shown to be carrier independent (Tsuji et al., 2007). A recent study has described the interaction between Tax and host histone methyltransferase SMYD3, where SMYD3 contributes to preferential Tax presence in the cytoplasm (Yamamoto et al., 2011). Likewise, a new study has identified RNF4-mediated Tax nuclear-to-cytoplasmic enrichment that results in increased NF-κB and decreased CREB-mediated Tax activity (Fryrear et al., 2012). Selective depletion of RNF4 was shown to inhibit nuclear-cytoplasmic shuttling of Tax typically induced by DNA damage.

### Extracellular Tax contributes to neuropathogenesis of HTLV-1

Far less is known about Tax oncoprotein secretion by HTLV-1 infected cells. Extracellular Tax has been coupled to ATL and HAM/TSP symptoms. In patients with diagnosed HAM/TSP, Tax has been shown to hyperstimulate the immune system and antibodies against Tax have been shown to cross-react with neuronal protein heterogeneous nuclear ribonuclear protein-A1 (Kubota et al., 2000; Levin et al., 2002a,b). More specifically, extracellular Tax studies have investigated the ability of HTLV-1 to cause demyelination and inflammation of cells from the central nervous system (CNS; Alefantis et al., 2005b). Studies have demonstrated cytokine release by microglial cells, in response to extracellular Tax treatment, express TNF-α, interleukin-6 (IL-6), and interleukin-1β (IL-1β; Dhib-Jalbut et al., 1994). In a parallel study, neuronal cells showed a similar response mechanism via the release of TNF-α (Cowan et al., 1997). HTLV-1 cytokine induction is known to be dose dependent, Tax specific, and lasting up to 8 h after stimulation (Dhib-Jalbut et al., 1994). Collectively, all these cytokines have been shown to induce oligodendrocyte dysfunction which in turn leads to neuron demyelination and other pathologies observed in HAM/TSP patients (Alefantis et al., 2005b).

HTLV-1 is also known to infect T-cells *in vivo*, and even though it can infect microglia and astrocytes *in vitro*, infected T-cells are the common sources of extracellular Tax as they infiltrate the CNS during neurological disease (Szymocha et al., 2000a,b,c). More recently, Tax has been shown to be colocalized with cytoplasmic organelles that are involved in the exocytotic pathway such as the Golgi complex and the endoplasmic reticulum (ER; Alefantis et al., 2005b). Additionally, time lapse video microscopy established cytoplasmic Tax movement in a manner coupled to microtubule-associated protein migration or that of secretory vesicles. Full-length Tax protein presence was confirmed in the culture medium of the Tax-transduced cells.

## Tax Dysregulates Cell Cycle Progression

Tax has been implicated as one of the lead HTLV-1 proteins that control cell cycle. The interaction of Tax with cell cycle components often results in dysregulation of the normal cell cycle controls leading to a number of cellular abnormalities including aneuploidy and immortalization of T-cells, all of which play key roles in oncogenesis.

### Tax disrupts cell cycle by inactivating check point proteins

By disrupting cell cycle check points, Tax prevents programmed cell death and promotes the proliferation of infected cells. This has obvious advantages in terms of viral proliferation and Tax carries out this function in a number of ways. Specifically, Tax is able to disrupt the G1/S transition which may be largely due to direct interaction of Tax with cyclin dependent kinase (Cdk) 4 and Cdk6 via its amino terminal domain (Neuveut et al., 1998; Schmitt et al., 1998; Haller et al., 2002; Yang et al., 2011). This causes Cdk proteins to interact with Cyclin D and, subsequently, to phosphorylate Retinoblastoma protein (Rb) in the Rb/E2F complex. This inactivates Rb and releases E2F, a protein involved in the regulation of the G1/S transition. Alternately, Tax can induce proteasomal degradation of Rb (Kehn et al., 2005). Tax also binds directly to Chk1 and Chk2 proteins leading to their inactivation (Haoudi et al., 2003; Park et al., 2004, 2006; Datta et al., 2007). This results in an unchecked interaction between Cdc25 and Cyclin B/Cdk1. Cyclin B/Cdk1 is responsible for regulating the G2/M checkpoint which, when it is stimulated by Cdc25, signals the cell to move forward into mitosis. Through interactions with other cellular proteins, Tax effectively modulates the rate of transition between various stages of the cell cycle. For example, Tax influences E2F and Cyclin B/Cdk1 to deregulate S phase and mitosis transitions in an accelerated manner. This increased rate of replication could result in errors throughout the cell cycle and thus promote cellular transformation. Moreover, Tax transactivates the transcription of proteins which promote cell growth such as Cyclin D2, Cdk4, and Cdk6 (Santiago et al., 1999; Iwanaga et al., 2008; Silbermann et al., 2008; Mizuguchi et al., 2009). Tax localizes at the centrosome during M phase (Pumfery et al., 2006; Afonso et al., 2007). Centrosomes operate as the MTOC of the cell in that they modulate the cells microtubule network. This network is critical for functions such as chromosome segregation, cell division, cellular development, and intracellular support (Pumfery et al., 2006; Afonso et al., 2007). Tax localization at this site during M phase suggests a key role for Tax to play in aneuploidy.

There appear to be three general types of errors that can occur leading to aneuploidy: (i) increased centrosome duplication; (ii) failure to complete cytokinesis; and (iii) incomplete or improper splitting during mitosis (Pumfery et al., 2006). Tax associates with hsMAD1, a mitotic spindle checkpoint (MSC) protein, causing MAD1 and MAD2 to translocate to the cytoplasm. By interfering with the localization of these two proteins, Tax effectively disrupts normal signaling in the event of a chromosomal segregation error. This allows M phase to proceed uninterrupted leading to aneuploidy (Jin et al., 1998; Pumfery et al., 2006).

A centrosomal protein known as Tax1BP2 has been implicated in Tax related aneuploidy. Studies have shown that cells with increased amounts of Tax1BP2 show a significantly lower tendency to contain supernumerary chromosomes and cells with diminished quantities of Tax1BP2 have a greater tendency toward aneuploidy (Ching et al., 2006; Pumfery et al., 2006). Furthermore, Tax interacts directly with Tax1BP2 and Tax mutants that fail to interact with Tax1BP2 show a decrease in aneuploidy (Ching et al., 2006; Pumfery et al., 2006). Collectively, these results indicate that Tax and Tax1BP2 perform opposite functions in HTLV-1 infected cells (Afonso et al., 2007). All together these observations provide evidence of a direct link between Tax expression and cell cycle abnormalities leading to cellular transformation.

### Tax directly regulates interleukin expression and promotes cellular proliferation and immune modulation

Studies of HTLV-1 have demonstrated the ability of the virus to promote the expression of cytokines and their receptors, such as T-cell growth factor interleukin-2 (IL-2) and the subunit IL-2 receptor α chain (IL-2Rα) of its receptor complex (Ballard et al., 1988; Ruben et al., 1988; Hoyos et al., 1989; McGuire et al., 1993; Good et al., 1996; Grassmann et al., 2005). The increased expression of these proteins leads to increased proliferation of infected cells. Furthermore, a number of other interleukins exhibit increased expression in cells infected with HTLV-1. Among them, IL-21 and its receptor subunit IL-21R stand out as IL-21 activates a number of intracellular pathways required for proliferation in T-cells. The recent work of Mizuguchi et al. has indicated that Tax specifically plays a role in the upregulation of IL-21 and IL-21R. In fact, these colleagues indicate not only an increase in IL-21 and IL-21R mRNA expression in the presence of Tax, but also that the promoters for each contain Tax responsive elements. It is important to note, however, that these experiments were conducted in Jurkat cells transfected with Tax and the induction of IL-21 by Tax has not yet been validated in an infected cell line (Mizuguchi et al., 2009).

In addition to IL-21, an upregulation of IL-13 and its receptor α1 is observed in HTLV-1 infected patients due to stimulation of the IL-13 promoter by Tax (Silbermann et al., 2008). IL-13 exhibits a number of immune modulating functions including inhibition of tumor immuno-surveillance. By overexpressing IL-13, HTLV-1 infected cells can overcome some of the host’s antiviral responses (Silbermann et al., 2008). Furthermore, Tax-mediated stimulation of the nuclear factor of activated T-cells (NFAT) pathway has been shown to be sufficient in order to induce IL-13; however, inhibition of the NFAT pathway did not abolish IL-13 upregulation. In fact, the work of Silbermann and colleagues shows that IL-13 can also be upregulated via Tax-mediated activation of the NF-κB pathway. This adds IL-13 to an increasing list of interleukins that are susceptible to upregulation via Tax-mediated stimulation (Niinuma et al., 2005; Silbermann et al., 2008; Mizuguchi et al., 2009). This collective observation could begin to explain the ubiquitous upregulation of interleukins in Tax expressing cells. The characterization of other interleukins that are regulated by Tax raises an interesting point for consideration. Mizuguchi and coworkers propose that the proliferation of HTLV-1 infected cells may be in response to several cytokines and not limited to IL-2. This would provide a possible explanation for the shift from IL-2-dependent to IL-2-independent growth patterns observed in infected cells as a combination of other Tax regulated cytokines could prove to be a stronger regulator of cellular growth than IL-2 alone. This avenue of thought will require further investigation to fully elucidate interleukin involvement in HTLV-1 infected cell growth. In the meantime, however, IL-2 remains a cytokine of significant interest.

One of the hallmarks of cellular transformation in HTLV-1 infected cells is the change from an IL-2-dependent to an IL-2-independent growth pattern. The recent work of Yoshita et al. (2012) has demonstrated that by activating the mTOR kinase, Tax can stimulate the mouse T-cell line, CTLL-2, to transfer from an IL-2-dependent growth to an IL-2-independent growth. This provides new evidence to the debate over how HTLV-1 transformed cells begin as IL-2-dependent and later become IL-2-independent. This debate has brought into question the oncogenic properties of Tax. For example, the work of Bellon et al. suggests that the oncogenic properties of Tax have been largely misunderstood. The authors observed that much of the experiments pertaining to Tax-mediated oncogenesis were carried out in established cell lines. They argued that cell lines contain their own idiosyncrasies and thus were not truly indicative of the cellular transformation that occurs in infected patients. Therefore, the authors performed a number of experiments in order to compare the effects of Tax on established cell lines as opposed to primary cells. Their results suggest that immortalization of primary cells by Tax alone is rare and that the expression of Tax is not sufficient to induce the cells to transition from IL-2-dependent growth to IL-2-independent growth (Bellon et al., 2010). This debate is ongoing and has led to postulations that Tax is important for the initiation of transformation thus suggesting another factor, for example microRNA, being responsible for chronic transformation (Jeang, 2010). Clearly, the full oncogenic properties of Tax remain to be elucidated.

Finally, the HTLV-1 basic leucine zipper factor (HBZ) has recently become a protein of interest in the HTLV-1 oncogenesis story. HBZ, an antisense viral protein, was originally identified as associated with CREB-2 (an antisense transcription factor similar to CREB) which inhibited viral transcription at the 5′ LTR (Gaudray et al., 2002). The work of Lemasson et al. (2007) demonstrated that HBZ could also interact with CREB and CBP/p300. This interaction effectively abolished binding to the TRE-1 as well as the CRE, thus silencing Tax-mediated transcription (Gaudray et al., 2002; Clerc et al., 2008). This transcription suppressor function is carried out by the HBZ protein; however, the HBZ mRNA has been shown to promote T-cell proliferation by Satou et al. (2006). Furthermore, it was shown that only spiced HBZ (as opposed to unspliced HBZ) could induce ATL cell proliferation (Yoshida et al., 2008). These observations combined strongly support the notion that HBZ plays an important role in HTLV-1 oncogenesis; however, further study will be required to fully determine the role of this interesting protein.

## Tax Promotes Cellular Transformation by Inhibition of Cellular DNA Repair Mechanisms

While the ability of Tax to directly immortalize cells remains a point of debate, the inhibition of cellular DNA damage repair mechanisms has been clearly demonstrated. The involvement of HTLV-1 Tax in DNA damage is twofold. Firstly, Tax interferes with a multitude of cellular DNA repair mechanisms, including base (BER) and nucleotide (NER) excision repair, human mismatch repair (MMR), non-homologous end joining (NHEJ), and damage response signaling via ATR/CHK1 (Jeang et al., 1990; Philpott and Buehring, 1999; Kao et al., 2000a; Lemoine et al., 2000; Haoudi et al., 2003; Marriott and Semmes, 2005; Edwards and Marriott, 2008; Ducu et al., 2011). In doing so, Tax effectively aids in perpetuating an environment that promotes the replication and maintenance of genomic lesions. HTLV-1 Tax impairs BER by targeting DNA polymerase β; the transrepression of DNA polymerase β promoter by Tax reduces the amount of enzyme available for repair of single stranded lesions of six nucleotides or less.

Though the NER functions to repair bulky mutations within DNA caused by UV damage and carcinogens, Tax suppresses this mechanism by targeting both the tumor suppressor p53 and proliferating cell nuclear antigen (PCNA). Interestingly, Schavinsky-Khrapunsky et al. (2008) report a phenomenon whereby low cellular Tax levels enhance NER, while elevated Tax levels within the cell impair NER. In cells expressing reduced levels of Tax, active p53 protein is greatly augmented and capable of stimulating NER. Though the levels of p53 mRNA and protein remain unaltered in cells expressing increased levels of Tax, the accumulated p53 is functionally inactivated and incapable of stimulating NER (Gatza et al., 2003; Schavinsky-Khrapunsky et al., 2008). Additional evidence suggests that increased levels of Tax impairs NER via a mechanism independent of p53 (Matsuoka and Jeang, 2011). Alternatively, the expression of Tax has been shown to transactivate the PCNA promoter, and interfere with binding of the repressor PIR (pirin, iron-binding nuclear protein) complex containing TBP at the PCNA promoter. Thus, the increased transcription of PCNA could function to impair NER (Kao and Marriott, 1999; Kao et al., 2000b; Lemoine et al., 2000). Protection against spontaneous and insertion/deletion mutations offered by the human mismatch repair is diminished by the expression of Tax. In examining the expression of MMR genes of patients with ATL, Morimoto et al. describe altered expression of genes including human MutL homolog 1 (hMLH1), human MutS homologs 2, 3, and 6 (hMSH2, hMSH3, and hMSH6), and human post-meiotic segregations 1 (hPMS1). They propose that the attenuated expression of hMSH2 and methylation within the hPMS1 promoter contribute to the malfunction of the MutS and MutL repair mechanisms (Morimoto et al., 2005).

NHEJ remains the most widely used cellular repair mechanism to correct double stranded DNA breaks in G0, G1, and S phase (Ducu et al., 2011). Tax targets the DNA-dependent kinase (DNA-PK) complex and more specifically the catalytic subunit (DNA-PK_CS_) and the kinase domain of DNA-PK, Ku, a dimer composed of Ku70 and Ku80 subunits (Ducu et al., 2011). Durkin et al. (2008) show that Tax colocalizes with active phosphorylated DNA-PK_CS_, which are more abundant in Tax expressing cells. By constantly activating DNA-PK_CS_, Tax appears to hijack and constitutively activate a normal DNA repair mechanism, ultimately impairing the ability of the cell to sense legitimate DNA damage (Durkin et al., 2008). Durkin and colleagues also show that Tax suppresses the expression of Ku80, ultimately decreasing the amount of active Ku within the nucleus. This diminished availability and function of Ku allows for the accumulation of unrepaired double stranded DNA breaks within the nucleus.

Tax has also been shown to target the DNA damage sensor ataxia telangiectasia mutated (ATM) and its target CHK2, both necessary for initiating the signaling involved in DNA repair of double stranded breaks (Reinhardt and Yaffe, 2009). Since ATM and CHK2 function to control the cell cycle progression through the G1/S checkpoint, targeting ATM and CHK2 by Tax prevents the appropriate sensing and correcting of DNA damage and promotes progression through this checkpoint. ATM, a serine-threonine kinase, is stimulated by double stranded DNA breaks and becomes activated by phosphorylation and subsequent dissociation into monomers (Reinhardt and Yaffe, 2009). The expression of Tax causes dephosphorylation of ATM, which inactivates the kinase and prevents the accumulation of ATM on chromatin surrounding the damaged area (Van et al., 2001; Haoudi et al., 2003; Chandhasin et al., 2008; Chlichlia and Khazaie, 2010). The expression of Tax resulted in a reduction of ATM-mediated phosphorylation of the pre-existing phosphorylated H2A.X (γH2A.X) and CHK2, preventing the accumulation of γH2A.X foci in response to DNA damage. Thus, the recruitment of effector proteins such as MDC1 and CHK2 was impaired in the presence of Tax (Haoudi and Semmes, 2003; Park et al., 2006; Gupta et al., 2007; Durkin et al., 2008; Ramadan et al., 2008). This effectively blocks both scaffold formation and the positive feedback loop involved in the repair of double stranded breaks. Tax expression has also been shown to interfere with other proteins involved in the ATM damage signaling cascade. Firstly, Tax expression has been shown to sequester CHK2, DNA-PK, BRCA1, and MDC1 into TSS (Fryrear et al., 2012). By preventing ATM-mediated CHK2 phosphorylation, Tax expression results in increased amounts of inactive CHK2. Tax also binds directly to CHK2 and specifically inactivates the kinase activity of this protein.

As indicated above, Tax is involved in DNA damage in two ways. Firstly, interference by Tax with cellular response to and repair of DNA damage allows for the propagation of genetic mutations. The second means by which Tax contributes to DNA damage stems from the ability of Tax to generate genomic mutations. Kinjo et al. (2010) report that the presence of Tax in human primary cells induces the production of reactive oxygen species (ROS), though the mechanism for this remains incompletely understood. They further conclude that an increase in phosphorylated H2A.X (γH2A.X), a marker of DNA damage, resulted from the expression of Tax (Kinjo et al., 2010). Finally, the Tax protein of HTLV-1 has also been shown to induce DNA damage in a replication-dependent manner. Boxus et al. (2012) presented data implicating Tax expression in increasing the amount of supplementary replication origins, as well as the function of Tax in inappropriately activating these origins via a CBP/p300 mechanism, potentially resulting in double stranded breaks which were indicated by the accumulation of γH2A.X foci. Interestingly, the accumulation of γH2A.X in response to DSBs demonstrated by Boxus et al. directly contrast the reports of Chandhasin et al. (2008) discussed above. Thus, further research is necessary to discern the role of Tax in generation and accumulation of γH2A.X foci.

In addition to altering the abundance and activation of replication origins, Majone and Jeang (2000) demonstrate that Tax expressing cells exhibited increased presence of DNA fragments containing unprotected 3′-OH ends within micronuclei, and propose that Tax suppresses a mechanism for stabilizing DNA ends. It has also been shown that Tax expressing cells exhibit increased frequency of micronuclei formation (Majone et al., 1993).

Currently, the role of Tax in telomere maintenance is widely debated. Bellon et al. (2006) reported that unstimulated ATL cells have shorter telomere lengths than uninfected cells, yet ATL cells express increased levels of active hTERT. Upon stimulation, T-cells expressing Tax suppressed transcription of hTERT (Zane et al., 2011). By transcriptionally repressing hTERT, Tax expression prevents the maintenance of telomeres, and potentially contributes to the destabilization of DNA ends in double stranded breaks or chromosomal fusions (Durkin et al., 2008). The findings of Gabet et al. (2003) lend further support to the ability of Tax to suppress hTERT activity by inhibiting hTERT transcription. Competition between Tax and c-Myc for binding within the hTERT promoter results in repression of hTERT. Zane et al. (2011) have reported that Tax induces transcription of TRF1, TRF2, and POT1, which also aid in the maintenance of shorter telomeres. Conversely, it has been reported that Tax induces the expression of hTERT in quiescent T-cells (Gabet et al., 2003). Here, these authors report Tax-mediated activation of the hTERT promoter in a cell cycle dependent manner. Interestingly, this group demonstrates Tax-mediated repression of the hTERT promoter in growing T-cells (Gabet et al., 2003). Thus, further research is required to further elucidate Tax-mediated modulation of hTERT and telomere maintenance.

The Tax oncoprotein has been implicated in chromatin dynamics due to Tax-mediated repression of transcription of replication-dependent histone genes, which could subsequently impact the regulation of host gene expression. Bogenberger and Laybourn (2008) propose that this Tax-mediated reduction of core and linker histone levels could present an additional mechanism for induction of genetic mutations. Additionally, Tax could impact chromatin remodeling through its interaction and inactivation of p53, a tumor suppressor that normally recruits HATs and HDACs. By inhibiting the function of p53, Tax expression causes inappropriate recruitment of HATs and HDACs, subsequently altering chromatin remodeling (Schavinsky-Khrapunsky et al., 2008).

## Tax Interacting Proteins as Novel Therapeutic Targets

Current prognosis for patients with acute ATL remains poor. An aggressive chemotherapy regimen such as VCAP-AMP-VECP is recommended (Tsukasaki, 2012). A combinatorial regimen of Zidovudine/INFa is thought to have promise and introduction of arsenic trioxide to the mix is suspected to have a more synergistic influence in inducing Tax proteolysis (Kchour et al., 2009; Tobinai, 2009; Tanosaki and Tobinai, 2010; Nasr et al., 2011; Tsukasaki, 2012; 1–5). However, as the status stands, most patients with ATL are not curable with current chemotherapy regimens alone. Allogenic stem cell transplantation is recommended in many cases. New therapeutic candidates that are undergoing clinical trials include a defucosylated humanized anti-CC chemokine receptor 4 monoclonal antibody (Tsukasaki, 2012). Below we discuss several novel possible therapeutic approaches as they relate to Tax.

### Targeting the activation of host signaling events is a novel therapeutic approach

Activation of the NF-κB signaling pathway, either in a Tax-dependent or a Tax-independent manner is an important component that contributes to cell proliferation, protection from apoptosis and onset of drug resistance in ATL cells (Horie, 2007; Qu and Xiao, 2011). Inhibiting the NF-κB cascade not only induces apoptosis in ATL cells but also reduces the number of HTLV-1 infected cells in virus carriers. Therefore, inhibition of the host NF-κB response to reverse these above-mentioned effects in ATL cells has been explored as a promising therapeutic route.

BAY 11-7082, a well established inhibitor of the NF-κB cascade, was found to induce apoptosis in both virus-infected cell lines and primary ATL cells (Mori et al., 2002). It was also found to be effective in preventing primary tumor growth and leukemic infiltration in an ATL mouse model (Dewan et al., 2003). Another NF-κB inhibitor that has been demonstrated to have *in vitro* and *in vivo* efficacy against ATL and HTLV-1 transformed cells is dehydroxymethylepoxyquinomicin (DHMEQ), a derivative of epoxyquinomicin. Treatment with DHMEQ induces apoptosis in ATL and HTLV-1 transformed cells (Ohsugi et al., 2005, 2006; Watanabe et al., 2005). Bortezomib, a well-documented proteasome inhibitor has also been found to be effective in inducing cell death in ATL cells (Satou et al., 2004). While bortezomib can be exerting other effects as a proteasome inhibitor, it also directly influences the NF-κB signaling cascade by preventing the degradation of IκBα which results in inhibition of nuclear translocation of effector subunits such as p65. This could interfere with induction of NF-κB dependent anti-apoptotic survival mechanisms, thus contributing to apoptosis of ATL cells. Additionally, Bortezomib (PS-341) treatment was shown to cause stabilization of IκBα as expected and down regulated expression of NF-κB -dependent anti-apoptotic genes (Nasr et al., 2005). BMS-345541, an NF-κB inhibitor was shown to down regulate IKKβ kinase activity in HTLV-1 infected cells and induce apoptosis (Agbottah et al., 2008). A p53 and NF-κB modulatory anti-cancer compound 9AA was also shown to dramatically decrease survival of HTLV-1 transformed cells (Jung et al., 2008). The authors showed that treatment of HTLV-1 transformed cells with 9AA resulted in an increase in p53 protein and activation of p53 transcription activity. Additionally, the data indicated that 9AA-induced cell death could be blocked by introduction of a p53 small interfering RNA, linking p53 activity, and cell death. Therefore, the authors suggest that normal function of p53 that would be otherwise suppressed by Tax can be restored upon 9AA treatment. With regards to NF-κB activation, the paper demonstrates that NFkB transcriptional activity is reduced upon 9AA treatment. Specifically, while some increase can be seen in nuclear accumulation of p65, phosphorylation of p65 on Ser 536 is reduced upon 9AA treatment. Along those lines, there was decreased phosphorylation of IκBα suggesting that the upstream activator kinase IKK may also be influenced by 9AA. This also correlated with decreased DNA binding and transcription activation by p65. Capsaicin, a modulator of NF-κB signaling, inhibited the growth of ATL cells mainly due to the induction of cell cycle arrest and apoptosis. Capsaicin treatment induced the degradation of Tax and upregulation of IκBα, therefore leading to decreased nuclear p65 and decreased anti-apoptotic gene expression (Zhang et al., 2003). Another interesting compound, Pyrrolidine dithiocarbamate (PDTC), an anti-oxidant, was also shown to induce apoptosis in ATL cells and HTLV-1 infected cells. It was interesting to note that the viral protein Tax inhibited PDTC-induced apoptosis (Arima et al., 2004). PDTC is a widely utilized dithiocarbamate inhibitor of the NFkB signaling cascade. While the exact mechanism of action of PDTC in inhibition of the NFkB cascade is currently unclear, it is suspected to either directly influence the degradation of the IkB inhibitory subunit and/or inhibit the cellular oxidative stress responses by means of its anti-oxidant function (Schreck et al., 1992; Zhang et al., 2011; Ding et al., 2012).

Although NF-κB has been heavily studied, a number of other signaling pathways have also been investigated. Here several examples are discussed. Geranylgeranylation of Rho family GTPases is an essential modification that is critical for multiple cellular functions such as cytoskeletal organization, transcription and cell cycle regulation during tumorigenesis. HTLV-1 transformed cells were found to be sensitive to geranylgeranylation inhibitors, such as GGTI-298, which not only decreased transcriptional activity of the viral LTR, but also decreased total Tax protein in infected cells (Edwards et al., 2011). Inhibition of the Jak/STAT signaling pathway by CP-690,550 was also found to be effective *in vitro* and *in vivo* against HTLV-1-induced ATL (Belrose et al., 2011). Inhibition of Cdks by drugs such as Purvalanol A inhibited activated viral transcription in HTLV-1 infected cells while it did not significantly influence either basal transcription from the HTLV-1 promoter or from promoters of cellular genes (Wang et al., 2002; Agbottah et al., 2008). Purvalanol A treatment also upregulated apoptosis in HTLV-1 infected cells as evidenced by increased caspase 3 activity. The work of Agbottah et al. (2008) has suggested that a combinatorial approach, in which inhibitors against multiple pathways (i.e., NF-κB and Cdk) are employed in tandem, may be more effective in combating HTLV-1 infection.

### HDAC inhibitors may control the spread of viral infection

As discussed above, the interplay between HDACs and Tax plays an important role in transcription regulation. Thus, HDACs provide a potential target for therapeutic treatment. Along these lines, Valproate (2-*n*-propylpentanoic acid, VPA), a histone deacetylase inhibitor (HDACi), triggers Tax expression, thereby exposing the latent HTLV-1 reservoir to immune destruction (Belrose et al., 2011). Additional HDACis such as MS-275, suberoylanilide hydroxamic acid (SAHA), and LBH589 also inhibited proliferation of HTLV-1 infected and primary ATL cells (Nishioka et al., 2008). MS-275 and SAHA treatment resulted in an alteration of the host cell cycle proteins and induced a larger population of HTLV-1 transformed cells to go into apoptosis. MS-275 additionally influenced the NF-κB signaling cascade by down regulating phosphorylation of IKK, decreased nuclear translocation of NF-κB subunits and decreased promoter binding (Nishioka et al., 2008). In the case of LBH589, it was shown that this HDACi induced hyperacetylation of non-histone proteins such as Hsp90 in addition to histones (Hasegawa et al., 2011). This hyperacetylation of Hsp90 resulted in a disruption of its chaperone function and decreased levels of phosphorylated Akt. LBH589 was shown to induce caspase-2-mediated apoptosis in HTLV-1 transformed cells. Additionally, LBH589 also resulted in a marked decrease in multiple host factors involved in ATL proliferation and invasion including CCR4 and IL-2R. Interestingly, it was also demonstrated that LBH589 suppressed HBZ expression thus adding weight to the more recent report on inhibition of HBZ-SI by shRNA resulting in cell growth inhibition in ATL cells (Zhi et al., 2011).

## HTLV-1 Shows a High Degree of Similarity to HTLV-2 and HTLV-3

HTLV-1 was the first discovered human retrovirus in the early 1980s. Soon after its discovery HTLV-2 was described, whereas HTLV-3 and -4 subtypes were isolated only recently (Calattini et al., 2005; Wolfe et al., 2005). HTLV-2 is shown to be less pathogenic, however, it is involved in the development of HAM/TSP (Tsubata et al., 2005). Moreover, recent studies have indicated that co-infection with HTLV-2 may confer an immunological benefit in HIV-1 infected patients, a stark contrast to HTLV-1/HIV-1 co-infections which have been shown to exhibit no effect at best (reviewed in Beilke, 2012). Finally, HTLV-3 and -4 have not yet been associated to any pathology, most likely because of their recent identification, low number of identified patients and lack of proper reagents. Furthermore, little characterization of HTLV-4 has been published; thus only HTLV-1, -2, and -3 are discussed in this review. All four types of HTLVs, HTLV-1 to HTLV-4 contain a transactivator protein Tax, namely Tax-1, Tax-2, Tax-3, and Tax-4.

### Each Tax exhibits a similar yet unique protein structure

**Figure 4:**
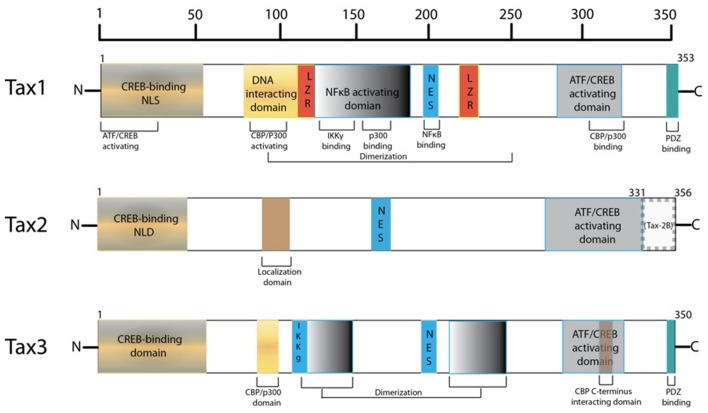
**Structural and functional domains of Tax1, Tax2 and Tax3**. Specific domains, and the regions involved in transcriptional activation pathways are indicated. Nuclear Localization Signal (NLS), nuclear export signal (NES), leucin zipper region (LZR), CBP/p300 binding sequence, NF-κB binding domain as well as PDZ binding motif are shown. Tax-2A has 331 amino acid (aa) residues, whereas Tax-2B has a 25 aa C-terminal extension, which is depicted in Figure 4 with a dotted square.

The protein structure of Tax-1, Tax-2, and Tax-3 is presented in Figure 4. Tax-1 and Tax-2 have various common domains. In the N-terminal region of Tax-1 lies a CREB-binding region, a zinc finger domain, and binding domains required for interaction with proteasomal subunits, transcriptional coactivators, and proteins involved in transcription, cell cycle progression, and in cell signaling regulations. The NLS is located within the first 60 amino acid of Tax-1, and similarly, a nuclear localization determinant (NLD) lies in the first 42 amino acids of Tax-2 (Sheehy et al., 2006; Turci et al., 2006). Within Tax-2 lies an additional localization domain of about 10 amino acids at position 90–100, which has been described to be involved in cellular localization (Meertens et al., 2004a). Meertens et al. (2004a) found that replacing aa 90–100 sequence in Tax-1 with that of Tax-2 redirects most of the chimeric protein into the cytoplasm as compared to predominantly in the nucleus in the case of wild type Tax-1. The PZD binding motif on the C-terminus of Tax-1 is absent in Tax-2. This motif allows Tax-1 to interact with many cellular factors, mostly scaffolding proteins, such as human disk large (hDlg), human homolog Scrib (hScrib), membrane-associated guanylate kinase-3 (MAGI-3), pro-IL16 and Erbin, all of which are unable to bind to Tax-2 (Lee et al., 1997; Rousset et al., 1998; Suzuki et al., 1999; Wilson et al., 2003; Hirata et al., 2004; Ohashi et al., 2004; Ress and Moelling, 2006; Arpin-Andre and Mesnard, 2007; Okajima et al., 2008; Higuchi and Fujii, 2009). In the central region of Tax-1, Tax-2, and Tax-3 lies two leucine zipper-like motifs at amino acids 116–145 and 213–248 (Tax-1 116RNGYMEPTLGQHLPTLSFPDPGLRPQNLYT145, 213LPTTLFQPARAPVTLTAWQNGLLPFHSTLTTPGLIW248; Tax-2 116RNGCLEPTLGDQLPSLAFPEPGLRPQNIYT145, 213LPTTMFQPVRAPCIQTAWCTGLLPYHSILTTPGLIW248; Tax-3 116RNNCLELTLGEQLPAMSFPDPGLRPQNIYT145, 213FPTTLFQPTRAPAVQAPWHTGLLPCQKEIATPGLIW248). In the central region of Tax-1 lie two leucine zipper-like motifs at amino acids 116–145 and 213–248. This region is necessary for interacting with protein dimerization, DNA-binding domains for proteins involved in histone methylation, cell cycle progression, and in cell signaling transduction (Shimotohno et al., 1985; Basbous et al., 2003a; Boxus et al., 2008; Bertazzoni et al., 2011). There is a conserved region between Tax-1, Tax-2, and Tax-3, putative KID-like domain [amino acids 81 to 95 (Tax-1 80QRTSKTLKVLTPPIT96; Tax-2 80QRTSRTLKVLTPPTT96 Tax-3 80QRTTRTLKVLTPPTT96)], which is necessary for binding to CBP/p300 (Calattini et al., 2006). However, there are sequence differences of Tax-3 compared to Tax-1 and Tax-2, such as a lysine residue at 85, which is crucial for Tax-1 to bind CBP/p300 (Hiramatsu and Yoshikura, 1986). In Tax-3 the CR2 binding region (amino acids 312–319) is slightly different than that of Tax-1 and Tax-2 (Tax-1 311YTNIPISL320; Tax-2 311YTNIPVSI320; Tax-3 311YSTVPFSL320), which has also been shown to be crucial for the ability of Tax-1 and Tax-2 to bind to CBP/p300 (Scoggin et al., 2001). The two amino acids belonging to the M22 domain are also different between Tax-1, Tax-2, and Tax-3 proteins (Tax-1 130TL131; Tax-2 130SL131; Tax-3130AM131; Smith and Greene, 1990). Tax3 also contains a PDZ binding motif at its C-terminus similar to Tax-1 (Calattini et al., 2006).

### Tax-1, Tax-2, and Tax-3 each interact with a different subset of cellular proteins

Tax-1 and Tax-2 have more than 75% homology of their amino acid sequences, and they also have similar functions in HTLV-infected cells, however Tax-1 may have a higher oncogenic potential compared to Tax-2 (Slamon et al., 1984; Seiki et al., 1986; Grassmann et al., 1992; Akagi and Shimotohno, 1993; Ross et al., 1996; Robek and Ratner, 1999; Kondo et al., 2006). Similar to Tax-1, Tax-2 also induces the expression of a number of cellular genes through its interaction with several transcription factors (Feuer and Green, 2005). Tax-3, on the other hand, displays between 26 and 30% divergence at the amino acid level from Tax-1 and Tax-2, respectively, and has been shown to be functionally more closely related to Tax-1 than Tax-2 (Calattini et al., 2006). Despite these differences, Tax-1 and Tax-2 both aid in transactivation of the viral promoter though the CREB/ATF and the NF-κB pathways.

The CREB/ATF family of transcription factors play a crucial role in the cell growth, survival, and apoptosis by modulating CRE-directed gene transcription in response to signals such as growth factors (Hai and Hartman, 2001; Persengiev and Green, 2003). *In vitro*, Tax-1 exhibits interactions with various proteins of the CREB/ATF family transcription factors, such as CREB, CREM, ATF1-ATF4, XBP1 (Franklin et al., 1993; Low et al., 1994; Bantignies et al., 1996; Winter et al., 2007; Boxus et al., 2008). Tax-2 proteins are also able to activate the CREB/ATF pathways, and can form a Tax-2-CREB/ATF-TxRE ternary complex *in vitro* by interacting with the b-Zip domain of CREB/ATF factors (Ross et al., 1997). The functional regions or domains important for transactivation through the CREB/ATF signaling pathway are similar, but not identical, in Tax-1 and Tax-2 (Ross et al., 1997). Due to the similarities in sequences of Tax-1 and Tax-3, Tax-3 also exhibits interactions with proteins from the CREB/ATF family made possible, through the presence of putative KID-like domain in Tax-3, which is critical for binding to CBP/p300 (Calattini et al., 2006; Chevalier et al., 2006).

Studies comparing the relative transactivation functions of Tax-1 and Tax-2 indicate significant differences as well as similarities in transactivation activities via NF-κB pathways between the Tax-1 and Tax-2 proteins (Sheehy et al., 2006). Both Tax-1 and Tax-2 activate the canonical NF-κB pathway through interacting with multiple NF-κB regulators. Although, the complete scheme of canonical NF-κB activation by Tax-1 and Tax-2 has not been fully elucidated yet, the activation of the IKK complex by Tax-1 and Tax-2 through binding with its scaffold subunit IKKγ (NEMO) is considered as a central event (Meertens et al., 2004b; Higuchi and Fujii, 2009). In addition to the canonical NF-κB pathway, Tax-1 activates the non-canonical NF-κB pathway (Xiao et al., 2001). Tax-1 binds to the IKK complex and NF-κB2/p100, resulting in IKKα mediated p100 phosphorylation and subsequent p100 processing into p52 (Xiao et al., 2001). Interestingly, Tax-2 cannot induce p100 processing into p52 when transiently expressed in the Jurkat T-cell line, although Tax-2 can activate the canonical NF-κB pathway to a level comparable to Tax-1 (Higuchi et al., 2007). The major defect of Tax-2 in p100 processing can be attributed to its inability to interact with p100 (Higuchi et al., 2007). Moreover, Tax-2 interaction with proteins such as IKKγ may account for its ability to increase transcription via the NF-κB pathway (Lewis et al., 2002).

### Tax-2 functions to inhibit cellular p53

A primary function of HTLV-1 Tax-1 is to inactivate the cellular p53 tumor suppressor in order to establish immortalization and transformation of T-cells. Consequently, high levels of cellular inactive, wild type p53 is found in Tax-transfected, HTLV-1 cell lines, as well as primary cells isolated from ATL patients. Interestingly, in HTLV-2 infected cells (both subtypes A and B), high levels of transcriptionally inhibited p53 are identified (Mahieux et al., 2000a,b). Tax-2A appears to inhibit p53 function less efficiently as compared to Tax-1 or Tax-2B (Mahieux et al., 2000a,b). Mechanistically, the inhibition of p53 by Tax-2 occurs through the activation of the NF-κB pathway, similar to Tax-1. This also imparts a lack of correlation to p53 inhibition by Tax-2 to CREB/ATF activation. Tax-2 also uniquely interacts with CBP, but not p300 in order to repress p53 (Meertens et al., 2004b).

### Tax-1, but not Tax-2 is a potent induced of micronuclei formation

Distinct phenotypic differences are observed in Tax-1 and Tax-2 expressing cells *in vitro*. This could explain why HTLV-2 is not associated with leukemic disease. Tax-2 has a lower transactivation activity of the HTVL-2 LTR, indicating a decrease in viral gene expression as well as a suppression of cellular Tax-2-responsive genes (Feuer and Green, 2005). Correlating with the enhanced transactivation effect of Tax-1, Tax-1 expressing cells induce micronuclei formation, which is a marker for genomic instability (Semmes et al., 1996; Xie et al., 2006). To date, Tax-2 has been shown to lack micronuclei inductive ability, which seems to correlate with a loss of the carboxy-termini as compared to Tax-1.

### Tax-1, Tax-2, and Tax-3 exhibit different patterns of localization

Tax-1 has been described to shuttle between the nucleus and cytoplasm being predominantly localized in the nucleus, where it is been shown to form speckled structures (Semmes and Jeang, 1996). However, Tax-2 is localized predominantly in the cytoplasm of the HTLV-2-immortalized or transformed T-cells due to the presence of a 10 amino acid domain (amino acids 90–100) unique to Tax-2 (Meertens et al., 2004a; Chevalier et al., 2005). Furthermore, by transiently transfecting HeLa cells with GFP-Tax-3_Pyl43_ (a full-length provirus), Calattini et al. (2006) showed that Tax-3 has a strong nuclear localization that is similar to that of Tax-1 and simian Tax-3 but different from that of Tax-2. Nevertheless, some cytoplasmic speckles are also detectable in Tax-3_Pyl43_-expressing cells (Calattini et al., 2006). A combination of the NLS and the NES contained within Tax-1, as well as the susceptibility of the protein to PTMs such as sumoylation and ubiquitination results in the shuttling and retention of Tax-1 in the nucleus and/or the cytoplasm. Indeed, Tax-2 has been shown to be modified by ubiquitination and sumoylation *in vitro* (Turci et al., 2009). Additionally, this Tax-2 was shown to be localized in the nucleus with RelA, indicating homologous *in vitro* function of Tax-1 (Turci et al., 2009).

### T-cell immortalization of Tax-1 versus Tax-2

Due to the fact that HTLV-2 does not cause leukemia as HTLV-1 does, it is important to determine the mechanism of T-cell immortalization of Tax-1 as compared to Tax-2. In one recent study, Tax-2 has been shown to immortalize human CD4 + T-cell at a higher activity than those transduced with Tax-1 (Imai et al., 2012). These distinct differences in T-cell immortalization may play a role in the drastically different pathogenesis associated with these two viruses. Another recent study has demonstrated the oncogenic properties of Tax-2 in the context of CD4 + T-cell immortalization. Ren et al. (2012) immortalized CD4 + T-cells to a CD3/TCRαβ/CD4/CD25/CD45RO/CD69 phenotype using Tax-2, therefore generating a distinct T-cell line. These cells constitutively activated PI3K/Akt, IκB/NF-κB, MAPK, and STAT3 (Ren et al., 2012).

### Characterization of HTLV-3 Tax-3

As the most recently discovered HTLV virus, there is a great need to characterize the pathogenicity of HTLV-3 as compared to HTLV-1. To date, no pathogenicity has been associated with HTLV-3, a virus which only has approximately 60% homology to HTLV-1. Early data indicates that Tax-3 displays strong similarities to Tax-1 in terms of sequence homology and motifs (Calattini et al., 2006). Tax-3 is able to activate transcription *in vitro* from the LTRs of HTLV-1, -2, and -3 as well as activates some cellular genes through the activation of the NF-κB pathway and inhibits transcriptional activity of cellular p53 (Calattini et al., 2006). A very recent study by Chevalier et al. (2012) utilized a DNA microarray to determine the global gene expression profile of different cell lines expressing Tax-1, Tax-2, or Tax-3. From this analysis, 48 common genes were found differentially regulated by all HTLV-1 transactivators screened, indicating that dysregulation of these genes are characteristic to all HTLV-1 infections. Additionally, this group identified 70 genes which are specifically upregulated by Tax-1 and Tax-3, indicating that these two viral proteins are closely related in terms of dysregulation of cellular genes. In contrast, Tax-1 and Tax-2 had only one gene in common as Tax-2 and Tax-3 only had eight genes in common (Chevalier et al., 2012). Functionally, the genes upregulated by both Tax-1 and Tax-3 are involved with regulation of transcription, apoptosis, NF-κB, immunity, proliferation, and differentiation. This is the first study to show that Tax-3 is a functional analog of Tax-1, suggesting a similar associated pathogenesis.

## Conclusion

The past 35 years have yielded a significant amount of meaningful data, not only in regard to HTLV-1, but in regard to the Tax oncoprotein itself. In this review, the role of Tax in major cellular events such as transcription, cell cycle, and DNA damage have been discussed. PTMs and subcellular localization have been shown to have both an effect on Tax as well as being affected by Tax. Numerous interactions have been highlighted which illustrate the oncogenic potential of this transactivator protein. Yet, it remains clear that, while well studied, the mechanisms of Tax as they relate to oncogenesis are still incompletely understood. While the labyrinth of interactions between Tax and host cellular proteins remains complex and inadequately illuminated, one simple fact is made starkly apparent: tax does not act alone.

This deceptively simple concept has driven, and should continue to drive, current and future research into this oncoprotein. Research into the interactions between Tax and other viral proteins (such as HBZ), interplay between Tax and miRNA during cellular transformation, and modulation of intricate cellular complexes (such as CBP/p300 and SWI/SNF) by Tax all provide promising avenues for further understanding of Tax-mediated cellular transformation requirements and, consequently, possible therapeutic targets. In fact, the promiscuity of Tax provides both great strength and weakness to the effectiveness of therapeutically targeting the oncoprotein in that direct targeting of Tax has a radiating effect on the major cellular pathways. It is important to note, however, that without a detailed understanding of the various branches of the Tax interactome, there exists the strong possibility of uncontrolled off-target effects. With improved understanding and more focused therapeutic approaches, modulating cellular Tax could have broad implications for the host cell and, consequently, be a first step in effective clinical treatments of HTLV-1 disease states.

Thanks to the excellent work of numerous talented researchers in the field, understanding the comprehensive role of Tax in HTLV-1 infections continues to be an evolving process. Many of the discoveries mentioned earlier in this review will provide promising avenues of future investigation. For example, a growing understanding of miRNA machinery has created another level of complexity in better defining cellular and viral protein regulation. Further research into the interactions between Tax and cellular miRNA (and possibly its machinery) and the resulting effect on oncogenesis will be required (Bellon et al., 2010; Jeang, 2010). This area of research naturally leads into investigations of the interactions between Tax and cellular miRNA and components such as Drosha and Dicer (Rahman et al., 2012). The study of this interaction may shed light on a number of the mechanisms of Tax as they relate to protein regulation and cell cycle. Moreover, the further development of animal models including rat, rabbit, and humanized mouse models will prove critical in advancing the understanding of the mechanisms of Tax *in vivo*. The development of such models would also advance the study of possible therapeutic targets for both viral and cellular genes unique to infected cells. Finally, more fundamental concepts will require further study, including the development of a crystal structure for Tax and further study into histone degradation versus mRNA downregulation as seen in Tax expressing cells. Cumulatively, much has been learned about Tax over the past 35 years, but there remains much more to discover before this fascinating oncoprotein can be fully understood.

## Conflict of Interest Statement

The authors declare that the research was conducted in the absence of any commercial or financial relationships that could be construed as a potential conflict of interest.
